# The Addition of Sprint Interval Training to Field Lacrosse Training Increases Rate of Torque Development and Contractile Impulse in Female High School Field Lacrosse Players

**DOI:** 10.3390/jfmk8030089

**Published:** 2023-06-24

**Authors:** T. Brock Symons, Alexandra H. Roberts, Kathleen A. Carter, John F. Caruso

**Affiliations:** 1 Department of Counselling, Health, and Kinesiology, Texas A&M University—San Antonio, San Antonio, TX 78224, USA; 2 Department of Dietetics, Nutrition and Sport, La Trobe University, Melbourne, VIC 3086, Australia; 3 College of Engineering, Science Technology, Agriculture, Central State University, Wilberforce, OH 45384, USA; 4 Department of Health and Sport Sciences, University of Louisville, Louisville, KY 40292, USA

**Keywords:** high-intensity interval training, skeletal muscle function, isometric strength, isokinetic concentric strength

## Abstract

Field lacrosse requires sudden directional changes and rapid acceleration/deceleration. The capacity to perform these skills is dependent on explosive muscle force production. Limited research exists on the potential of sprint interval training (SIT) to impact explosive muscle force production in field lacrosse players. The purpose of this study is to examine SIT, concurrent to field-lacrosse-specific training, on the rate of torque development (RTD), contractile impulse, and muscle function in female high school field lacrosse players (n = 12; 16 ± 1 yrs.). SIT was performed three times per week, concurrent to field-lacrosse-specific training, for 12 weeks. Right lower-limb muscle performance was assessed pre-, mid-, and post-SIT training via isometric and isokinetic concentric knee extensor contractions. Outcomes included RTD (Nm·s^−1^), contractile impulse (Nm·s), and peak torque (Nm). RTD for the first 50 ms of contraction improved by 42% by midseason and remained elevated at postseason (*p* = 0.004, effect size (ES) = −577.3 to 66.5). Contractile impulse demonstrated a training effect across 0–50 ms (42%, *p* = 0.004, ES = −1.4 to 0.4), 0–100 ms (33%, *p* = 0.018, ES = 3.1 to 0.9), and 0–200 ms (22%, *p* = 0.031, ES = −7.8 to 1.6). Isometric (0 rad·s^−1^) and concentric (3.1 rad·s^−1^) strength increased by 20% (*p* = 0.002, ES = −60.8 to −20.8) and 9% (*p* = 0.038, ES = −18.2 to 0.0) from SIT and field-lacrosse-specific training, respectively (*p* < 0.05). SIT, concurrent to field-lacrosse-specific training, enhanced lower-limb skeletal muscle performance, which may enable greater sport-specific gains.

## 1. Introduction

Women’s field lacrosse is a global sport with a total of 29 nations competing at the highest level [1]. Its popularity within the United States of America continues to grow with 96,762 female high school participants and 3028 high schools, competing in women’s field lacrosse in 2022 [2]. With the popularity of women’s field lacrosse growing at the club, high school, university, and national levels, it is important for athletes, coaches, and healthcare professionals, such as athletic trainers and physical therapists, to explore and develop appropriate training strategies to optimize performance in women’s field lacrosse.

As a sport, field lacrosse demands a high degree of coordination, agility, and speed from its players, as performance includes rapid changes in direction, continuous activity, transitions between acceleration and deceleration, and multiple bouts of intermittent high-intensity sprints [3]. Studies investigating sprint-related workloads during a college field lacrosse game for female players found that they can cover a distance of 656 m at speeds greater than 15 km·h^−1^, reach maximum speeds ranging from 24.1 to 26.2 km·h^−1^, and experience high-intensity acceleration counts between 51 and 177 during a game [4,5]. The capacity to perform these skills during competition and/or training requires superior lower-limb skeletal muscle strength and an enhanced ability to generate force rapidly [6,7]. Sprint exercises are an extensively used training approaches throughout Europe, the United States, Australia, and many other countries [8] due to their association with greater sports performance [9,10]. They are employed by training and coaching staff to increase skeletal muscle function and improve high-speed movements [11]. Research demonstrated sprint training significantly improves skeletal muscle power output; maximal 10, 20, and 30 m sprint speed; and linear acceleration [6,9,10,12]. Taylor et al. [6] proposed that improvements in skeletal muscle power and sprint speed likely resulted from enhancements in contractile properties and further adaptations to the lower-limb extensor muscles via the repeated production of rapid high-force contractions associated with sprint training [10,13,14,15].

Rapid force/torque production is an important characteristic of human skeletal muscle function and is vital to successful sports performance [16,17]. A high rate of force/torque development is a significant contributor to the performance of rapid and forceful movements [17]. Rapid and forceful movements require athletes to contract their skeletal muscles over short time intervals, which may not permit the generation of maximal skeletal muscle force to be attained [18,19]. The rate of force/torque development is a common parameter used to measure skeletal muscles’ capacity to generate force/torque and is obtained from the force– or torque–time curves during explosive/rapid isometric contractions [16,17]. Research has indicated that rapid force/torque generation may be a more sensitive determinant of acute and chronic adaptations to the neuromuscular system than maximal voluntary contractile force following strength training [16,20,21,22]. Research to date has shown significant improvements in the rate of force/torque development following various modes of resistance training [23]. Traditional machine-based and free-weight resistance training programs, in addition to explosive dynamic and static (isometric) resistance training programs, significantly enhance the rate of force/torque development [24,25,26]. 

To date, no study to our knowledge has examined the effect of sprint interval training (SIT) on skeletal muscle function, as assessed by the rate of torque development, contractile impulse, and skeletal muscle strength in young female field lacrosse players. If effective as a means of enhancing skeletal muscle function, and in combination with its known benefits in aerobic fitness, SIT may be a valuable and efficient training strategy for the sport of field lacrosse. Further, SIT may enable athletes to realize optimal gains in more than one facet of field lacrosse performance during a single training session [27,28]. Thus, the current study aimed to determine the effects of SIT, in combination with field lacrosse-specific training on lower-limb rate of torque development, contractile impulse, and skeletal muscle function in female high school field lacrosse players. It is hypothesized SIT plus field-lacrosse-specific conditioning would improve rate of torque development, contractile impulse, and skeletal muscle strength. To test this hypothesis, we exposed participants to SIT three times per week for 12 weeks and assessed their lower-limb skeletal muscle performance via isokinetic concentric dynamometry.

## 2. Materials and Methods

### 2.1. Participants and Study Design

Fourteen healthy female high school field lacrosse players (age: 16.0 ± 0.9 yrs., height: 161.2 ± 5.3 cm, mass: 61.9 ± 11.2 kg, body mass index: 23.9 ± 4.5 kg/cm^2^, percent body fat: 27.5 ± 6.5) volunteered to participate. Inclusion required the participants to be active members of the female varsity high school field lacrosse team. Players were informed of the purpose of the research study and were instructed that declining to participate would not affect their selection to the team or influence their playing time. To control for physical maturation, each subject completed the Self-Administered Pubertal Stage Survey, which is consistent with physician-conducted pubertal staging and provides a maturation score similar to those of the Tanner scale [29,30]. Participants were free of any lower-limb musculoskeletal or neuromuscular limitations and had not incurred a severe lower-limb or hip fracture within the past three years. Informed written parental consent and participant assent were obtained prior to participation, and all experimental procedures were approved by the Human Subjects Research and Institutional Review Board.

Participants served as their own control, as no control group (i.e., field-lacrosse-specific conditioning only) was employed in the current study. As identified by Chapman et al. [31] it would be unethical to not allow all players to participate fully in every training session across the preseason and competitive season. Participants were familiarized with all testing procedures prior to data collection. Further, all participants completed three testing sessions: preseason, midseason, and postseason. Isometric and isokinetic concentric knee extensor testing was performed on the participant’s dominant lower limb. The study timeline spanned 15 weeks and included both the preseason (three weeks) and competitive season (nine weeks), with the participants scheduled to take part in 33 training sessions. Two participants were unable to complete the study due to injury and illness. Therefore, an overall total of 12 female high school field lacrosse players completed the study.

### 2.2. Procedures

#### 2.2.1. Isometric and Isokinetic Testing

The Biodex Quick Set System 3 dynamometer (Biodex Medical Systems, Shirley, NY, USA) was used for all isometric and isokinetic strength testing with a sampling frequency of 100 Hz [32,33,34]. All strength tests were analyzed using the Biodex Advantage Software, version 3.2. The Biodex was calibrated in accordance with the manufacturer’s specifications before the start of each testing session. Each participant was seated in an upright position in the Biodex chair with a seat back angle of ~85°. To limit extraneous body movements all participants were stabilized via two shoulder straps that crossed the participant’s chest, a waist strap, and a thigh strap. The lateral femoral epicondyle was aligned with the center of the dynamometer shaft to establish the axis of rotation around the knee joint. The participant’s range of motion was then determined by having them completely extend (0°) their lower limb and slowly return it to a comfortable position slightly past 90° flexion. To negate the influence of gravity, the lower limb was weighted according to the manufacturer’s specifications. The participant’s lower limb was fully extended to maximize the gravitational effect, at which point the Biodex Advantage software established gravity correction. This correction was then applied to all strength measurements.

Before the start of isometric and isokinetic concentric knee extensor testing, a five-minute warm-up was performed on a stationary cycle ergometer at ~50 rpm against one kilopond of resistance. The sequence of testing was standardized for all participants with isometric torque at 0 rad·s^−1^ (5 s hold, knee angle ~90°) and assessed first, followed by isokinetic concentric torque, which was randomized for angular velocity (1.57 rad·s^−1^ and 3.14 rad·s^−1^) across all participants. The participants performed six maximal knee extensor contractions at all angular velocities and were instructed to kick as fast and as hard as possible [18,35,36], and a ten-second rest was given between each contraction, during which time the lower limb was passively returned to the starting knee angle of ~95°. A 60 s rest period was given between each isometric contraction to compensate for the longer contraction duration (5 s) [37,38]. A two-minute rest period was given between each angular velocity. Outcome measures included peak isometric and isokinetic knee extensor concentric torque (Nm) with the highest value of the six maximal repetitions performed used for all data and statistical analysis.

#### 2.2.2. Data Analysis

All rapid torque–time variables were determined from the peak isometric torque–time curve utilizing a customized Microsoft Excel 2016 spreadsheet (Microsoft, Redmond, WA, USA) [39,40]. Rapid torque–time outcome measures included maximum isometric knee extensor torque at 50, 100, and 200 ms from the onset of contraction (ISOM_50_, ISOM_100,_ and ISOM_200_, respectively) [41]. The rate of torque development (RTD) was quantified as the linear slope of the isometric torque–time curve (ΔTorque/ΔTime) at the following time intervals: 0–50, 0–100, and 0–200 ms from the onset of contraction (RTD_0–50_, RTD_0–100_, and RTD_0–200_, respectively) [41,42]. These time intervals were designated to represent early (RTD_0–50_) and late (RTD_0–200_) torque–time characteristics and are believed to characterize distinct physiological parameters [41,42]. Contractile impulse across the above-mentioned time intervals (0–50 ms, IMP_0–50_; 0–100 ms, IMP_0–100_; 0–200 ms, and IMS_0–200_) was quantified as the area under the torque–time curve (∫Torque ΔTime) [43,44]. The onset of contraction was established as the moment the isometric torque produced by the participant equaled 4 Nm [42,43].

### 2.3. Training

#### 2.3.1. Sprint Interval Training

Sprint interval training (SIT) took place over 12 weeks, during which participants were required to train three times per week, and consisted of maximal sprints of varying distances. The total possible number of training sessions was 33 (3 sessions lost to midseason testing). SIT was performed on an outdoor grass playing field when weather permitted and in a gymnasium during inclement weather. During training, sprint times were not recorded. However, encouragement was provided to participants during all SIT tasks by both the coaching staff and their peers. Although the rate of perceived exertion was not monitored, participants were encouraged to push themselves to maintain a rating of between 9 and 10 on the Borg Scale (0–10) throughout the duration of the SIT training. The participants continued their field-lacrosse-specific training with the number of practices and games varying from three to five, and one to three per week, respectively.

Sprint interval training consisted of three separate training sessions (SIT Session 1, SIT Session 2, and SIT Session 3). Sprint interval training Session 1 included maximal sprints (10–14 repetitions) at a distance of 40 m separated by 15–20 s of active recovery (walking or jogging). Session 2 involved two-three sets of all-out 15 m sprints with sets comprising 10 to 12 repetitions. Fifteen seconds of rest (no walking or jogging) was provided between repetitions and 120 s of active recovery was utilized between sets. Lastly, SIT Session 3 required the participants to perform three to four suicides (16 m, 25 m, 50 m, 75 m) with a 60 s rest between repetitions. All SIT sessions were administered by the same member of the research team.

#### 2.3.2. Field-Lacrosse-Specific Training

Field lacrosse-specific training included a brief warm-up, aerobic conditioning, basic field lacrosse skill development, tactical drills, and scrimmages (Table S1). Scheduling of practices and games followed the State High School Athletic Association calendar.

### 2.4. Statistical Analysis

Data are presented as means ± standard deviations. Statistical analysis was performed using SigmaStat version 3.5 (Systat Software, Inc. San Jose, CA, USA). A Kolmogorov–Smirnov test was used to determine data normality. Results of the Kolmogorov–Smirnov tests required the following outcome measures to be assessed with non-parametric analyses: ISOM_50_, ISOM_100_, isokinetic knee extensor concentric torque at 3.14 rad·s^−1^, and RTD_0–50_. All other outcome measures were determined to be normally distributed; thus, parametric analyses were used. Changes in peak torque and rapid-torque–time variables were analyzed using a one-way repeated measures ANOVA with one within-group factor (time: preseason, midseason, and postseason). If the normality test failed (*p* < 0.05), Friedman repeated measures ANOVA was performed on the ranks. Secondary analysis was performed using pair-wise multiple comparison procedures with a Tukey correction. The magnitude of the responsiveness to the SIT between preseason and midseason and preseason and postseason was determined using Cohen’s d effect size statistic. Effect sizes were classified as small (d < 0.19), moderate (d = 0.20–0.49), medium (d = 0.50–0.79), large (d = 0.80–1.09), and very large (d > 1.10) [45]. The significance level was established at *p* < 0.05.

A one-tailed paired sample means that power analysis was performed to confirm an appropriate sample size to detect a significant difference (*p* = 0.05) in maximal isometric torque, our most important and variable outcome measure. As one group was examined over time (pre-, mid-, and post-intervention), it is reasonable to assume that a one-tailed hypothesis is appropriate because a training program (and hence the mid- and post-intervention values) is expected to elicit a large improvement. Based on a power of 0.80, a mean difference of 15 Nm, and a standard deviation of 20 Nm, a sample size of 13 was required to achieve the desired power. Despite losing two participants, resulting in a total n of 12, a statistical power above 0.80 was observed for the change in maximal isometric knee extensor torque (β = 0.93).

## 3. Results

### 3.1. Isometric Muscle Strength and Rate of Torque Development

The maximum isometric knee extensor torque demonstrated a 10% increase at the midseason time point and a 20% increase following 12 weeks of training (main effect for time, *F*(2,22) = 8.878, *p* = 0.002). Post hoc analysis revealed that only the postseason increase in mean isometric torque of 31.7 N·m was significantly different from the preseason value (Table 1). Isometric knee extensor torque at 50, 100, and 200 ms also exhibited a training response; however, only ISOM_100ms_ and ISOM_200ms_ demonstrated a significant main effect for time (*Χ^2^*(2) = 8.909; *p* = 0.012) and (*F*(2,22) = 3.662; *p* = 0.044), respectively. In both cases, the isometric knee extensor torque significantly increased by the midseason assessment (ISOM_100_ ms, +33.3% (*p* < 0.05) and ISOM_200ms_, +22.2% (*p* < 0.05)).

**Figure 1:**
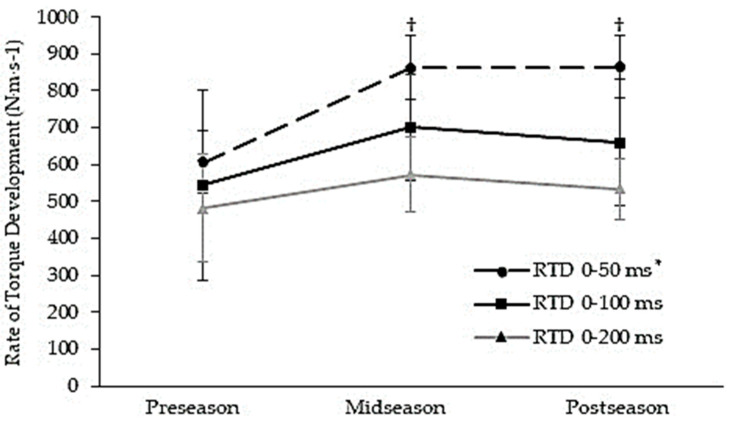
Mean (± standard deviation) change in rate of torque development (RTD) following 12 weeks of combined sprint interval training (SIT) and field-lacrosse-specific training. * *p* < 0.05, significant time effect across the 12-week training period. † *p* < 0.05, a significant difference from the preseason. ms = Milliseconds.

Figure 1 shows the mean change in the rate of torque development (RTD) for the time intervals of 0–50 ms, 0–100 ms, and 0–200 ms following 12 weeks of combined SIT and field-lacrosse-specific training. The RTD from the onset of contraction across the first 50 ms of contraction (RTD_0–50ms_) increased by 42.4% (*Χ^2^*(2) = 11.091; *p* = 0.004) following training. Significant increases in RTD_0–50ms_ were observed at both the midseason and postseason time points (preseason: 607.3 ± 451.1 vs. midseason: 862.7 ± 292.7, *p* < 0.05; and preseason: 607.3 ± 451.1 vs. postseason: 864.7 ± 255.4, *p* < 0.05). The rate of torque development over the first 100 ms and 200 ms of contraction increased by +21% (*p* = 0.051) and by +11% (*p* = 0.076) across the 12 weeks of combined SIT and field-lacrosse-specific training, respectively.

### 3.2. Contractile Impulse

**Figure 2:**
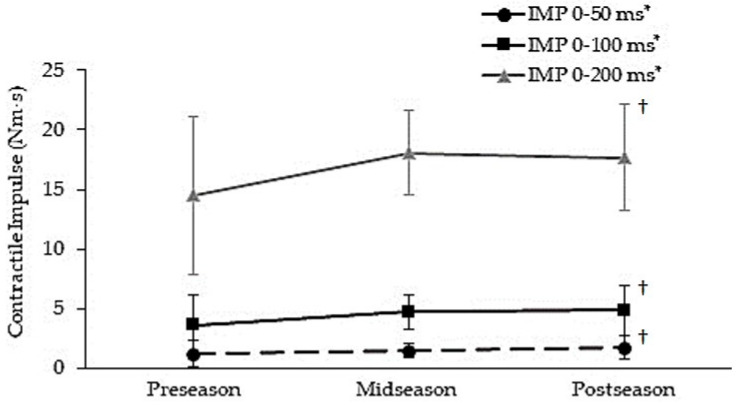
Mean (± standard deviation) change in contractile impulse (IMP) following 12 weeks of combined sprint interval training (SIT) and field lacrosse specific-training. * *p* < 0.05, significant time effect across the 12-week training period. † *p* < 0.05, a significant difference from the preseason. ms = Milliseconds.

Figure 2 compares the change in contractile impulse over the 12-week combined training period from the onset of contraction to 50 ms, 100 ms, and 200 ms. Contractile impulse demonstrated a significant training effect across all three selected time intervals (IMP_0–50ms_: *F*(2,18) = 4.679, *p* = 0.025; IMP_0–100ms_: *F*(2,18) = 4.679, *p* = 0.018; and, IMP_0–200ms_: *F*(2,18) = 4.372, *p* = 0.031). Contractile impulse increased from 1.3 ± 1.1, 3.7 ± 2.6, and 14.5 ± 6.6 N·m·s (preseason) when obtained at time intervals of 0–50, 0–100, and 0–200 ms, respectively, to 1.8 ± 1.0, 4.8 ± 2.1, and 17.6 ± 4.4 N·m·s (postseason), respectively (*p* < 0.05) following 12 weeks of SIT and field-lacrosse-specific training.

### 3.3. Isokinetic Skeletal Muscle Strength

Peak isokinetic concentric knee extensor torque (1.57 rad·s^−1^) did not change over the 12-week combined SIT and field-lacrosse-specific training period (Table 1). However, when isokinetic concentric knee extensor torque was performed at 3.14 rad·s^−1^, a significant training effect was observed (*Χ^2^*(2) = 6.545; *p* = 0.038). Post hoc analysis demonstrated the mean increase in peak isokinetic concentric knee extensor torque following the 12-week combined SIT and field-lacrosse-specific training period was significantly greater than the mean preseason value (*p* < 0.05).

## 4. Discussion

The present study aimed to determine the effects of SIT, in addition to field-lacrosse-specific training, on skeletal muscle performance. It was hypothesized that SIT combined with field-lacrosse-specific training would improve the rate of torque development, contractile impulse, and skeletal muscle strength. The results of the study indicate the early (0 to 50 ms) rate of torque development was significantly improved at both midseason and postseason time points following the completion of 12 weeks of SIT and field-lacrosse-specific training. Contractile impulse also demonstrated a significant training effect across all three selected time intervals IMP_0–50ms,_ IMP_0–100ms,_ and IMP_0–200ms_ (range of increase from 22% to 42%). Furthermore, our findings also revealed a significant increase in isometric (0 rad·s^−1^) and isokinetic concentric (3.14 rad·s^−1^) knee extensor peak torque following 12 weeks of SIT in combination with field-lacrosse-specific training.

The ability to rapidly generate skeletal muscle force/torque is a key performance indicator and is of paramount importance to success in many sports. Sprint intervals, in combination with field-lacrosse-specific training, increased contractile RTD across the first 50 ms by 42.4% (*p* = 0.004) and by 21% (*p* = 0.051) over the first 100 ms of contraction. Direct comparisons with previous studies investigating the effects of exercise training on contractile RTD must be made with caution as training mode, intensity, movement velocity, training duration, and participants’ age and sex differ between studies [23]. Tillin et al. [26] and Oliveira et al. [23] both explored the effects of four and six weeks of isometric strength training on knee extensor rate of force development in young adult males. Tillin et al. [26] concluded 16 sessions (over four weeks) of explosive isometric training demonstrated a significant increase in explosive force measured at 50, 100, and 150 ms from the onset of contraction. Oliveira et al. [23] found 18 sessions (over six weeks) of isometric strength training designed to enhance explosive and maximal strength increased the rate of force development during the early phase (0–20 ms) of contraction. The Denadai group further explored the effects of fast-velocity concentric (3.14 rad·s^−1^) [46] and eccentric (3.14 rad·s^−1^) [47] isokinetic resistance training on the rate of force development in young male adults. The rate of force development after six weeks of fast-velocity concentric isokinetic resistance training demonstrated a significant improvement in the rate of force development at 0–10 ms up to 0–90 ms [46]. Likewise, eight weeks of fast-velocity eccentric isokinetic resistance training improved the rate of force development during the early phase (0–50 and 50–100 ms) of contraction [47]. Mangine et al. [48] employed an eight-week high-intensity isotonic resistance training program (3–5 repetitions at 90% of participants 1 RM) in strength-trained males and determined the rate of force development significantly improved over the first 50 ms of contraction. Taken together, these results illustrate the potential of high-intensity resistance training of various modes and its positive influence on the contractile rate of force development. It is of importance and of practical significance to note the current study observed similar results in early phase contractile rate of force development as the above-mentioned studies utilizing a simpler and more convenient form of training.

Kinnunen and colleagues [49] further demonstrated the potential and convenience of sprint interval training when they investigated its ability to induce neuromuscular adaptations, changes in force production, and on-ice performance in female ice-hockey players. High-intensity interval training was implemented as an extension of the player’s on-ice training [49]. The sprint training occurred two times per week and entailed six, 30 s all-out uphill running sprints. Kinnunen et al. [49] found the rate of force development, as determined as the steepest initial force increase during the early phase of a maximum voluntary isometric contraction, was significantly improved by two-and-half weeks of uphill sprint training. Our results are in agreement and further, demonstrate the merits of sprint interval training in addition to field-lacrosse-specific training as a viable and convenient method to increase the contractile rate of force over the early phase of contraction (<100 ms).

The mechanism to explain the increase in early phase contractile rate of force after sprint interval training has not been elucidated. It is conceivable the all-out sprint training employed in the current study provided a time-specific stimulus (i.e., <100 ms) that enhanced the participants’ abilities to generate skeletal muscle force during the initial 100 ms of contraction. During fast lower-limb movements, as with those seen during sprinting, contraction times (i.e., foot contact time where body mass acts as the main resistance) characteristically range between 50 and 250 ms [18,50]. Thus, the rapid and short contractions performed during the repeated stance phases of the SIT employed in the current study may have provided an appropriate training stimulus to increase the rate of force development of the knee extensors during the initial 100 ms of contraction. Additionally, the absolute intensity of an exercise bout dictates the motor unit recruitment profile and it was proposed sprint training is a mode of training that actively recruits type II skeletal muscle fibers [51,52]. Therefore, it is plausible that SIT is effective at improving the rate of force development because it recruits, and thus enhances, the structural and metabolic profile of type II skeletal muscle fibers that are situated higher in the recruitment hierarchy [51,52]. Liljedahl et al. [52] demonstrated that four weeks of sprint training (on a cycle ergometer) in young adult females significantly increased the cross-sectional area of type II muscle fibers, especially the type IIX muscle fibers. Furthermore, Methenitis and colleagues demonstrated changes in the early rate of force development correlated positively to changes in the cross-sectional area of type IIX muscle fibers [53]. Therefore, the possibility of selectively activating type II motor units (characterized by rapid force production) during the repeated stance phases of the sprint running exercise may have provided sufficient stimulus to alter the force-generating capacity of the knee extensors during the current study.

As specified by Aagaard and colleagues [18], the contractile impulse is one of the most important strength parameters as it integrates the aspect of contraction time, which allows for the examination of the contraction’s temporal characteristics. The capacity to develop a large contractile impulse is an essential characteristic for any athlete and, specifically, field lacrosse players. During practice and game situations, players continually have to outsmart opponents with unexpected movements by rapidly changing their speed and direction during offensive and defensive possessions [54]. Sports related movements require abrupt and deliberate changes in speed and direction (i.e., velocity), and any change in velocity is governed by the generated contractile impulse [54]. In the present study, contractile impulse exhibited a significant training effect across all three selected time intervals (IMP_0–50ms_, 42%; IMP_0–100ms_, 33%; and, IMP_0–200ms_, 22%). Our results are in agreement with Aagaard et al. [18], who found 14 weeks of heavy-resistance training in young male adults increased contractile impulse across the following time intervals: 0–30, 0–50, 0–100, and 0–200 ms. Further, Suetta et al. [44] examined the effects of a 12-week unilateral lower-limb rehabilitation program in combination with resistance training in older adults. Resistance training significantly increased contractile impulse for the following time intervals 0–30 (32%), 0–50 (32%), 0–100 (28%), and 0–200 ms (27%). Caution should be employed when comparing the present study’s contractile impulse findings with previous investigations examining the contractile impulse response to training as exercise mode, intensity, and duration as well as participant ages and sex differ from one study to another. Nevertheless, the results of the current study illustrate the potential of SIT, in addition to sport-specific training, to significantly increase contractile impulse through the early phase of skeletal muscle contraction. This is of practical importance to athletes and training/coaching staff since the ability to accelerate, decelerate, and change direction are key components of the sport of interest and all of these acts necessitate the ability to generate well-time and directed contractile impulses [54].

Improvements in skeletal muscle strength, as assessed by maximal isometric and isokinetic concentric contractions, following sprint interval training have been inconsistent and, at best, demonstrated non-uniform results across different angular velocities [27]. Sokmen et al. [55] found that ten weeks of sprint interval training with active recovery in recreationally active young male and female adults elicited no change in isometric knee extension torque. However, Kinnunen and colleagues [49] who utilized two-and-a-half weeks of high-intensity interval training consisting of 30 s of all-out uphill sprints in female ice hockey players, found a significant improvement in maximal isometric plantar flexor strength. This result is in agreement with the present study’s finding that maximum isometric knee extensor torque displayed a significant increase (20%) following 12 weeks of training. Kinnunen et al. [49] concluded the improvement in isometric test performance after sprint training was the result of increased neural drive to the agonist muscles and reduced coactivation of the antagonist’s muscles. It is not inconceivable to consider that the repeated stance phases under load (body weight) offered by the sprint training utilized in the current study provided a sufficient stimulus to induce similar neuromuscular adaptations to those observed in the initial weeks of resistance training and witnessed in the Kinnunen et al. [49] study.

Isokinetic concentric strength at 1.57 and 3.14 rad·s^−1^ was employed to assess dynamic skeletal muscle strength in the present study. Peak isokinetic concentric knee extensor torque (1.57 rad·s^−1^) showed no improvement over the 12 weeks of combined SIT and field-lacrosse-specific training period and is in agreement with Ferley et al. [56] and Sokmen and colleagues [55]. Ferley et al. [56] examined the effects of incline and flat-surface HIIT comprised of 12 interval and 12 continuous running sessions across six weeks in well-trained young adult runners and demonstrated no improvement in the average knee extensor torque at 1.57 rad·s^−1^. Like Ferley et al. [56] and the present study, Sokmen et al. [55] found no enhancement of isokinetic concentric knee extensor torque at 1.05 rad·s^−1^. Taken together, these results suggest that sprint interval training performed at high intensities is not an effective training mode to increase dynamic skeletal muscle strength at slow angular velocities. 

In contrast to the outcome observed at the slow angular velocity, SIT in addition to field lacrosse-specific training induced a significant increase in isokinetic concentric knee extensor torque when performed at 3.14 rad·s^−1^. This finding is supported by Sokmen and colleagues [55] who demonstrated that ten weeks of SIT yielded a significant improvement in isokinetic knee extension at 5.24 rad·s^−1^. In support of Sokmen et al. [55] and the current study’s results, Freely et al. [56] found a significant increase in the average knee extensor peak torque at both 3.14 rad·s^−1^ and 5.24 rad·s^−1^ after six weeks of incline and flat-surface HIIT. Furthermore, Ozgunen et al. [57] examined the isokinetic concentric knee extensor strength in well-trained youth soccer players following eight weeks of repeat sprint training. Isokinetic concentric knee extensor strength at 3.14 rad·s^−1^ and 4.19 rad·s^−1^ improved significantly following the eight-week sprint training. Taken together, these results imply a potential for SIT to influence isokinetic concentric torque at angular velocities greater than 3.14 rad·s^−1^. However, caution should be employed when interpreting the above results as Ferley et al. [56] concluded that non-uniform benefits occurred across the right and left lower-limbs during isokinetic skeletal muscle testing, and the improvement in average peak torque at both angular velocities (3.14 rad·s^−1^ and 5.24 rad·s^−1^) was also observed in their control group who maintained a weekly running program [56]. Further research is needed to fully elucidate the impact of high-intensity sprint interval training on skeletal muscle torque at angular velocities greater than 3.14 rad·s^−1^.

The absence of a control group performing field-lacrosse-specific training is the only limitation in the current study. A control group was not employed in the current study because it would have required some players to receive less training than their counterparts across the preseason and competitive season, and this was deemed unethical [31]. The absence of a field-lacrosse-specific training group raises the question; would the field-lacrosse-specific training alone provide enough training stimulus to significantly enhance lower-limb skeletal muscle performance?

One study that did explore the seasonal changes induced by field lacrosse practice and competition over a six-week field lacrosse season in middle school (7th and 8th grade) girls resulted in improved overall fitness (number of sit-ups and push-ups, increased long jump, and reduced 1.5-mile run time) [58]. Data pertaining to seasonal changes in lower-limb skeletal muscle performance, specifically in regard to RTD and contractile impulse employed in the current study, among female high school field lacrosse players are currently unavailable. However, observed seasonal changes in lower-limb skeletal muscle performance of young female athletes from different sports may provide some insight into the potential adaptive stimulus of sport-specific training. Pereira et al. [59] concluded that eight weeks of in-season volleyball-specific-only training for high school girls (age: 14 years) did not result in significant changes in skeletal muscle strength or countermovement jump performance. Furthermore, Siegler et al. [60] concluded that 10 weeks of in-season soccer-specific training resulted in nonsignificant improvements in shuttle running, vertical jump, twenty-meter sprint, and Wingate performance among high school girl soccer players (age, 16 years). Lastly, Meszler and Vaczi [61] who studied adolescent girls (age: 16 years) undergoing basketball-specific training only (control group) during a seven-week competitive season showed no significant changes in countermovement jump height, agility, balance, and maximal isokinetic concentric knee extensor torque (at 1.05 rad·s^−1^) [61]. However, Meszler and Vaczi also demonstrated that basketball-specific training led to a significant increase of approximately 17% in maximal isokinetic concentric knee extensor torque at 3.14 rad·s^−1^ [61]. Meszler and Vaczi’s findings suggest that the 9% increase (*p* = 0.038) in maximal isokinetic concentric knee extensor torque at 3.14 rad·s^−1^ observed in our study may be attributed to field-lacrosse-specific training alone. However, their study findings reveals significant inconsistencies within and between their control and experimental groups.

Caution should be exercised when interpreting the Meszler and Vaczi reported finding for maximal isokinetic concentric knee extensor torque at 3.14 rad·s^−1^. The basketball-specific training group did not show any significant improvements in any other outcome measures related to lower-limb skeletal muscle performance, such as maximal isokinetic concentric knee extension (1.05 rad·s^−1^) and flexion (1.05 rad·s^−1^ & 3.14 rad·s^−1^), countermovement jump height, agility (T-sprint & Illinois agility test), and balance measures [61]. Moreover, the experimental group that underwent lower-limb high-intensity plyometric training (double- and single-limb hurdle and lateral core jumps, single-leg forward hop, and double-leg depth jump) in addition to basketball-specific training, did not show significant improvements in maximal isokinetic concentric knee extensor or flexion at 1.05 rad·s^−1^ and 3.14 rad·s^−1^, agility, or balance (except for improved countermovement jump height, *p* < 0.05) [61]. The non-uniform improvement in measures of lower-limb skeletal muscle performance is somewhat surprising. Both the control and experimental groups performed the same basketball-specific training, yet the control group demonstrated a ~17% improvement in maximal isokinetic concentric knee extensor torque at 3.14 rad·s^−1^, whereas the experimental group displayed an ~8% improvement. This discrepancy is further confounded by the experimental group performing a total of 131 jumps (repetitions) across two sessions/week for the first three weeks and a total of 184 jumps (repetitions) across two sessions/week throughout weeks four through six during the high-intensity plyometric training, in addition to the basketball-specific training. With the added mechanical loading experienced by the knee extensor muscles during the high-intensity plyometric training, in addition to the mechanical loading of basketball-specific training, it is reasonable to hypothesize that maximal isokinetic concentric knee extensor torque gains would be superior to those achieved with basketball-specific training alone. Casting uncertainty on the reported finding for maximal isokinetic concentric knee extensor torque at 3.14 rad·s^−1^ found in the Meszler and Vacz study.

Taken together, these findings suggest significant improvements in skeletal muscle performance observed in the present study may reflect the addition of SIT to traditional field-lacrosse-specific training and not solely the result of field-lacrosse-specific training.

Similar observations have been found in male high school field lacrosse players. Rosene [62] found that ten weeks of field lacrosse practice and competition increased one-repetition maximum (1 RM) leg press and leg extension in male high school (age: 16 years) field lacrosse players (control group). However, when lower-limb resistance training was performed in addition to field-lacrosse-specific training and competition (experimental group), improvements in 1-RM leg press and leg extension were significantly greater when compared to the field-lacrosse-specific training and competition-only group [62]. Thus, field lacrosse training and competition alone can increase measures of skeletal muscle performance. However, the inclusion of another mode of training (resistance training) in addition to field-lacrosse-specific training significantly improved leg press and leg extension by 1-RM when compared to the field-lacrosse-specific-training-only group [62]. Furthermore, Talpey et al. [63] found that that the maximal isokinetic concentric knee extensor torque at 1.05 rad·s^−1^, 3.14 rad·s^−1^, and 5.24 rad·s^−1^ did not change from preseason to postseason testing in male field lacrosse players (age: 20 years) over a 16-week collegiate lacrosse season.

Collectively, these findings indicate that the notable enhancements in skeletal muscle performance observed in the present study may be due to the incorporation of SIT into traditional field-lacrosse-specific training, rather than solely the result of field-lacrosse-specific training.

A further limitation of the current study is the small sample size. Therefore, the findings of this research study cannot be generalized beyond female high school field lacrosse players. Additionally, given the young age of the participants (age: 16 years), it is possible that any form of training stimulus could lead to improvements in this group of athletes, regardless of the specific training program utilized. This idea has been challenged by the findings of Costa et al. [64], who showed that maximal knee extensor torque and lean mass did not naturally increase in young female swimmers as they progressed from ages 10 to 20 years.

## 5. Conclusions

This study revealed SIT, in combination with field-lacrosse-specific training, significantly improved the early phase rate of torque development (<50 ms) and contractile impulse across the first 200 ms of contraction. It is of practical significance to athletes, training staff, and coaching staff that the current study observed these results utilizing a simple and efficient form of training. Furthermore, we demonstrated that SIT and field-lacrosse-specific training increased static and high-velocity dynamic knee extensor strength. Our results suggest that SIT, in addition to field-lacrosse-specific training, enhances aspects of lower-limb skeletal muscle performance and thus may enable athletes to realize optimal gains in more than one facet of field lacrosse performance during a single training session. Finally, this study helps fill the research gap on female high school lacrosse by providing additional data on female athletes participating in this sport.

## Figures and Tables

**Table 1:** Knee extensor peak torque, rate of torque development, and contractile impulse across the 12-week training period.

		Preseason	Midseason	Postseason	*p*-Value	ES; 95% CI
*Peak Torque (Nm)*						
Isometric	0 rad·s^−1^	157.9 ± 26.2	174.1 ± 36.0 (+10%)	189.6 ± 21.1 † (+20%)	0.002 *	1.1; −60.8 to −20.6
	ISOM_50_	31.6 ± 22.5	43.1 ± 14.6 (+36%)	41.7 ± 12.1 (+32%)	0.060	
	ISOM_100_	53.4 ± 23.7	71.2 ± 14.6 † (+33%)	64.9 ± 14.3 (+22%)	0.012 *	0.9; −34.5 to −1.1
	ISOM_200_	95.0 ± 26.3	116.1 ± 18.9 † (+22%)	105.9 ± 14.9 (+11%)	0.044 *	0.9; −40.5 to −1.7
Concentric	1.57 rad·s^−1^	120.2 ± 13.5	124.4 ± 13.7 (+4%)	122.5 ± 14.1 (+2%)	0.669	
	3.14 rad·s^−1^	92.7 ± 11.5	97.5 ± 9.6 (+5%)	101.1 ± 10.2 † (+9%)	0.038 *	0.8; −18.2 to 0.0
*Rate of Torque Development (Nm·s^−1^)*						
Isometric	RTD_0–50_	607.3 ± 451.1	862.7 ± 292.7 ‡ (+42%)	864.7 ± 255.4 † (+42%)	0.004 *	0.7; −577.3 to 66.5
	RTD_0–100_	545.2 ± 257.2	700.5 ± 144.9 (+28%)	659.4 ± 171.2 (+21%)	0.051	
	RTD_0–200_	482.6 ± 144.5	573.5 ± 100.2 (+19%)	535.3 ± 82.4 (+11%)	0.076	
*Contractile Impulse (Nm·s)*						
Isometric	IMP_0–50_	1.3 ± 1.1	1.5 ± 0.6 (+15%)	1.8 ± 1.0 † (+38%)	0.025 *	0.5; −1.4 to 0.4
	IMP_0–100_	3.7 ± 2.6	4.8 ± 1.4 (+30%)	4.9 ± 2.1 † (+32%)	0.018 *	0.5; 3.1 to 0.9
	IMP_0–200_	14.5 ± 6.6	18.1 ± 3.6 (+25%)	17.6 ± 4.4 † (+21%)	0.031 *	0.6; −7.8 to 1.6

Values are presented as means (± standard deviation). Percentage changes from preseason values shown in parentheses for each time point. * *p* < 0.05, significant time effect across the 12-week training period. † *p* < 0.05, a significant difference from the preseason, and ‡ *p* < 0.05, a significant difference from midseason. ES = Effect size, CI = confidence interval, ISOM = isometric, RTD = rate of torque development, and IMP = contractile impulse.

## Data Availability

The data will be made available by the corresponding author upon reasonable request.

## Supplementary Materials

The following supporting information can be downloaded at: https://www.mdpi.com/article/10.3390/jfmk8030089/s1, Table S1. Description of sprint interval training and field lacrosse-specific training.
