# Supplementary material for: The Addition of Sprint Interval Training to Field Lacrosse Training Increases Rate of Torque Development and Contractile Impulse in Female High School Field Lacrosse Players
Source: J Funct Morphol Kinesiol. 2023 Jun 24;8(3):89. doi: 10.3390/jfmk8030089 (PMC10366844; doi:10.3390/jfmk8030089)
Supplement: Supplementary file 1 [file jfmk-08-00089-s001.zip › jfmk-2327794-supplementary.pdf]

Supplemental Table

Supplemental Table S1. Description of sprint interval training and field lacrosse-specific training.

**Sprint Interval Training**			
	**Monday**	**Wednesday**	**Friday**
Weeks 1 through 4	40m sprint with 10 repetitions<br>20 seconds of passive rest	2 sets of 15m sprints with 10 repetitions<br>15 seconds of passive rest between repetitions<br>2 minutes of passive rest between sets	3 suicides (16, 25, 50, 75 m, touch line with foot)<br>1 minute of passive rest between each repetition
Weeks 5 through 8	40m sprint with 12 repetitions<br>20 seconds of passive rest	3 sets of 15m sprints with 10 repetitions<br>15 seconds of passive rest between repetitions<br>2 minutes of passive rest between sets	3 suicides (16, 25, 50, 75 m, touch line with foot)<br>1 minute of passive rest between each repetition
Weeks 9 through 12	40m sprint with 14 repetitions<br>20 seconds of passive rest	3 sets of 15m sprints with 12 repetitions<br>15 seconds of passive rest between repetitions<br>2 minutes of passive rest between sets	4 suicides (16, 25, 50, 75 m, touch line with foot)<br>1 minute of passive rest between each repetition

Field Lacrosse-specific Training

Fundamental / Skill Development

- Passing and catching drills (e.g. line passing, four corners)
- Ground ball drills (e.g. line drills, 1 verse 1)
- Shooting drills (both stationary and in motion)
- Position specific drills:
    - Attack player specific drills focusing on passing, catching, dodging, ground balls, and shooting
    - Midfield player specific drills focusing on passing, catching, dodging, ground balls, and defensive stick / foot work
    - Defensive player specific drills focusing on passing, catching, ground balls, and defensive stick / foot work

Team Offense and Defense Development

- 3 verses 2 and 3 verses 3 games

- 6 verses 5 and 6 verses 6 games
- situational scrimmages

m = meters and e.g. = example.
